# Fertilization differences alter the diversity and function of tea plant rhizosphere soil microbial communities, thus affecting tea plant growth

**DOI:** 10.3389/fmicb.2025.1708146

**Published:** 2025-11-14

**Authors:** Qi Zhang, Songhan Guo, Yulin Wang, Yankun Liao, Xiaoli Jia, Bitong Zhu, Qiqi Weng, Haibin Wang, Jianghua Ye

**Affiliations:** 1 College of Tea and Food Science/Fujian Key Laboratory of Big Data Application and Intellectualization for Tea Industry (Wuyi University), Wuyi University, Wuyishan, China; 2 College of Life Science, Longyan University, Longyan, China; 3 College of Life Sciences, Fujian Normal University, Fuzhou, China

**Keywords:** fertilization patterns, tea plants, characteristic microorganisms, microbial functions, tea plant growth

## Abstract

**Introduction:**

Fertilization is one of the main agronomic measures in tea plantation management, but the effects of different fertilization patterns on the structure, function, and growth of tea plant rhizosphere soil microbial communities are still unclear.

**Methods:**

This study compared the effects of three fertilization treatments: 100% chemical fertilizer (CF), 50% chemical fertilizer + 50% organic fertilizer (COF), and 100% organic fertilizer (OF) on tea plant growth and rhizosphere soil microorganisms. Plant growth indicators were measured, and soil microbial communities were analyzed via high-throughput sequencing of 16S rDNA and ITS regions. Microbial functions were predicted using FAPROTAX and FUNGuild.

**Results:**

The COF treatment resulted in the most significant promotion of tea plant growth, with the highest chlorophyll content (30.47 SPAD), leaf area (18.76 cm^2^), and hundred-bud weight (18.22 g). Soil microbial analysis revealed that while COF treatment significantly increased microbial biomass carbon (151.05 mg/kg) and phosphorus (105.56 mg/kg), CF treatment was more conducive to accumulating microbial biomass nitrogen (130.47 mg/kg). High-throughput sequencing indicated that COF treatment exerted the strongest impact on bacterial community structure, while OF treatment enhanced the migration rate and α diversity of both bacteria and fungi. Different fertilization treatments primarily altered the abundance of key microorganisms *Opitutus* and *Coccocarpia*, thereby influencing microbial fermentation and lichenized functions. Specifically, COF increased the abundance of *Coccocarpia* and reduced the abundance of *Opitutus*, thereby promoting soil lichenized functions while reducing fermentation functions.

**Discussion:**

The synergistic effect of combined chemical and organic fertilization enhances lichenized function and suppresses fermentation, which in turn improves the accumulation of soil microbial biomass carbon, nitrogen, and phosphorus, ultimately fostering tea plant growth. These findings suggest that the COF fertilization strategy can be further optimized and provide a theoretical basis for scientific fertilization management in tea plantations.

## 1 Introduction

Tea plants (*Camellia sinensis*) are a crucial cash crop in China, and their growth tightly linked to soil fertility and microbial communities (Hu et al., 2022). Fertilization, a key measure in tea plantation management, directly affects soil microecological balance, nutrient conversion, and tea plants' nutrient absorption and utilization (Miao et al., 2024). While long-term chemical fertilizer use can boost short-term tea yields, it induces leads to soil acidification, reduces microbial diversity, and degrades ecosystem (Zhang et al., 2025). In recent years, with the development of ecological agriculture, organic fertilizers have attracted significant attention as alternatives to chemical fertilizers, given their potential to improve soil structure, enhance microbial activity, and promote sustainable production (Bergstrand, 2022). Therefore, investigating how rhizosphere soil microorganisms respond to different fertilization patterns is critical for optimizing tea plantation fertilization strategies, facilitating tea plant growth, and increasing tea yields.

Soil microorganisms play a central role in nutrient cycling, with their community structure and function directly influencing soil fertility and plant growth (Hartmann and Six, 2023). For instance, long-term application of chemical fertilizers significantly alters nitrogen cycling processes in tea plantation soils, promoting nitrification driven by ammonia-oxidizing bacteria while enhancing denitrification activity, leading to nitrogen loss in forms such as N_2_O and exacerbating environmental risks (Dincă et al., 2022). Additionally, excessive application of chemical fertilizers can affect phosphorus availability, intensify microbial competition for phosphorus, reduce soil phosphorus mineralization capacity, and consequently impair tea plants' phosphorus uptake (Liu et al., 2021). In contrast, the application of organic fertilizers can increase soil organic matter content, promote the proliferation of beneficial microorganisms, and improve soil microenvironments (Ye et al., 2022). However, the slow nutrient release of organic fertilizers may fail to fully meet the rapid growth needs of tea plants (Shaji et al., 2021). Therefore, the combined application of organic and inorganic fertilizers is considered a potential strategy to balance soil nutrient supply and microbial activity (Bai et al., 2022). However, systematic research on how the combined application of organic and chemical fertilizers influences tea plant growth by regulating soil microbial communities and their functions remains lacking.

In recent years, the application of high-throughput sequencing technology has provided new insights into the structure and function of soil microbial communities (Lahlali et al., 2021). Numerous studies have shown that different fertilization patterns significantly influence the diversity and abundance of bacteria and fungi in tea plantation soils (Yan et al., 2022; Lei et al., 2024). For example, long-term application of chemical fertilizers alone reduces bacterial community diversity, while the use of organic fertilizers enhances microbial community stability and functional diversity (Han et al., 2021). Additionally, fertilization patterns affect the distribution of key functional microorganisms, thereby regulating soil nutrient transformation efficiency (Dincă et al., 2022). Wan et al. (2021) found that the combination of organic and chemical fertilizers is beneficial for improving the physical and chemical properties of citrus planting soil, promoting citrus growth, and increasing yield. Jin et al. (2022) found that the combination of organic and chemical fertilizers can increase bacterial diversity in lettuce rhizosphere soil, reduce fungal diversity, improve soil microbial community structure, and enhance lettuce yield and quality. It can be seen that the combination of organic and chemical fertilizers has a significant impact on plant growth. However, current research on how the combined application of chemical and organic fertilizers synergistically affects the functional networks of tea plant rhizosphere microorganisms remains limited, particularly regarding the mechanisms linking microbial diversity and function to tea plant growth under different fertilization regimes. Therefore, systematically investigating the effects of different fertilization patterns on the structure, function, and key characteristic microorganisms of tea plant rhizosphere microbial communities is crucial for elucidating the mechanisms by which soil microecology regulates tea plant growth.

Based on this, this study uses tea plants as the research subject, applying different fertilization patterns to the tea plants, and analyzes the effects of these fertilization patterns on tea plant growth. Additionally, high-throughput sequencing technology is employed to determine the bacterial and fungal communities in the rhizosphere soil of tea plants, analyzing the effects of different fertilization patterns on the structural composition of bacterial and fungal communities in the rhizosphere soil. Characteristic microorganisms that significantly distinguish between different fertilization patterns are screened and their functions are analyzed. Based on this, the study further analyzes the association between characteristic microorganisms and their functions and tea plant growth, aiming to reveal how fertilization patterns influence tea plant growth by altering soil microbial community structure and function. This research provides a theoretical basis for scientific fertilization in tea plantations and lays the foundation for microbial regulation in sustainable tea production.

## 2 Materials and methods

### 2.1 Experimental design and sample collection

The field experiment for this study was conducted in the Jinguanyin (*Camellia sinensis*) tea plantation located in Xibian Village, Xingcun Town, Wuyishan City, Nanping City, Fujian Province (27°39′43.37” N, 117°53′33.57” E). The soil type of this tea plantation is acidic red soil, with the following basic physical and chemical properties: pH value of 4.21, total nitrogen, total phosphorus, total potassium, available nitrogen, available phosphorus, available potassium, and organic matter content of 6.75 g/kg, 0.79 g/kg, 1.62 g/kg, 2.01 mg/kg, 4.41 mg/kg, 127.97 mg/kg, and 2.07 g/kg, respectively. The experimental design included three different fertilization methods: 100% chemical fertilizer (CF), 50% chemical fertilizer + 50% organic fertilizer (COF), and 100% organic fertilizer (OF). Each treatment had three independent replicates, with each replicate covering a tea plantation area of 100 m^2^. The chemical fertilizer used was a compound fertilizer (N: P_2_O_5:_ K_2_O = 1: 1: 1), with a recommended application rate of 0.07 kg/m^2^. The organic fertilizer was purchased from Zhongnong Lvkang (Beijing) Biotechnology Co., Ltd. in Beijing, China. It is a plant humus organic fertilizer (N: P_2_O_5:_ K_2_O = 3.5: 3: 3, organic matter content ≈ 45%), with a recommended application dosage of 0.30 kg/m^2^. Fertilizer application was conducted in October 2024 using the trench burial method. Specifically, a trench 20 cm wide and 25 cm deep was dug 30 cm from the main stem of the tea plant, and the required fertilizer for each treatment was uniformly spread into the trench before being covered with soil. Sample collection was conducted in May 2025. First, tea plant growth indicators were measured, followed by the collection of rhizosphere soil using the five-point sampling method (Miao et al., 2024). The rhizosphere soil sampling method involved randomly selecting five tea plants, removing ~25 cm of surface soil until reaching the tea plant roots, and collecting the soil adhering to the surface of the tea plant roots, which constituted the rhizosphere soil. The collected soil was thoroughly mixed to form one replicate. Each fertilization treatment had three independent replicates. The collected rhizosphere soil was used for soil microbial indicator measurements and high-throughput sequencing analysis of bacteria and fungi.

### 2.2 Measurement of tea plant growth indicators

The measurement of tea plant growth indicators included chlorophyll content, leaf area, and hundred-bud weight, with each indicator having three independent replicates. Chlorophyll content was measured using a chlorophyll analyzer (TYS-N, Beijing, China). For each replicate, the second-to-last leaf was randomly selected from eight tea plants, and the average value was calculated. Leaf area measurement also involved randomly selecting the second-to-last leaf from 8 tea plants, measuring the length and width of the leaf, calculating the leaf area as length × width × 0.7, and taking the average as one replicate. Hundred-bud weight measurement involved taking 100 standard bud tips (three leaves and one bud) from each replicate, weighing them, and using the total weight as the hundred-bud weight for that replicate. All measurements were conducted in three independent replicates.

### 2.3 Determination of soil microbial biomass carbon, nitrogen, and phosphorus

The determination of microbial biomass carbon, nitrogen, and phosphorus in tea plant rhizosphere soil was performed using the chloroform fumigation extraction method with reference to Gao et al. (2022). Briefly, the determination of microbial biomass carbon involved digesting the extract with a potassium dichromate-sulfuric acid mixture, measuring the absorbance at 350 nm, and converting the absorbance to its content. The determination of microbial biomass nitrogen involved reacting the extract with indophenol reagent, measuring the absorbance at 570 nm, and calculating the content. The determination of microbial biomass phosphorus involved neutralizing the extract with HCl solution, adding sulfuric acid-molybdenum antimony reagent for color development, measuring the absorbance at 882 nm, and calculating the content.

### 2.4 High-throughput sequencing analysis of soil microorganisms in the rhizosphere of tea plants

Total DNA from the rhizosphere soil of tea plants was extracted using the E.Z.N.A. Soil DNA Kit (Omega Bio-tek, Inc., USA), and the quality and concentration of the DNA were determined using a Nanodrop 2000 spectrophotometer (ThermoFisher Scientific, Inc., USA). Bacterial 16S rDNA was amplified by PCR using the universal primers 338F (5′-ACTCCTACGGGAGGCAGCAG-3′) and 806R (5′-GGACTACHVGGGTWTCTAAT-3′). Fungal ITS rDNA was amplified using the universal primers ITS1 (5′-CTTGGTCATTTAGAGGAAGTAA-3′) and ITS2 (5′-TGCGTTCTTCATCGATGC-3′) for PCR amplification.

The PCR amplification system and reaction protocol for bacteria and fungi were based on the method described by Jia et al. (2024). In brief, the total PCR amplification system was 25 μL, including 2 μL of DNA template (30 ng), 1 μL each of 5 μM forward and reverse primers, 3 μL of 2 ng/μL BSA, 12.5 μL of 2x Taq Plus Master Mix, and 5.5 μL of ddH_2_O. The PCR reaction protocol for bacteria was as follows: pre-denaturation at 95°C for 5 min, followed by 28 cycles (denaturation at 95°C for 45 s, annealing at 55°C for 50 s, and extension at 72°C for 45 s). The PCR reaction program for fungi is as follows: pre-denaturation at 94°C for 30 s, followed by 40 cycles (denaturation at 94°C for 5 s, annealing at 55°C for 15 s, and extension at 72°C for 45 s). PCR amplification products were purified using Agencourt AMPure XP magnetic beads (Beckman Coulter, Inc., USA) and sequenced using the NEB Next Ultra II DNA Library Prep Kit (New England Biolabs, Inc., USA). The constructed libraries were sent to Beijing Allwegene Technology Co., Ltd. (Beijing, China) for paired-end sequencing (PE250/PE300) on the Illumina MiSeq/NextSeq 2000/Novaseq 6000 platform (Illumina, Inc., USA). Raw sequencing data were subjected to bioinformatics analysis as described in Table 1, and based on the obtained OTUs, the potential functions of bacterial and fungal communities were predicted using the FAPROTAX and FUNGuild functional prediction tools, respectively.

**Table 1:** Bioinformatics analysis workflow and methods for sequencing data.

**Analysis process**	**Bacteria 16S**	**Fungus ITS**
Data splitting	The raw data was divided into different samples according to the barcode sequence.	
Data splicing	Use Pear (v0.9.6) software to filter and splice raw data. The sequences were removed from consideration if they contained ambiguous bases N, and cut out the parts with low quality score (≤20) in the sequences. During splicing, the minimum overlap setting was 10 bp, and the *p*-value setting was 0.0001.	
Filter and remove chimeras	After splicing, Vsearch (v2.7.1) software was used to remove sequences with length less than 230 bp and removed the chimeric sequence by uchime method according to the Gold Database.	After splicing, Vsearch (v2.7.1) software was used to remove sequences with length less than 230 bp (ITS2 was 230 bp) and removed the chimeric sequence by uchime method according to the Unite Database.
OTU clustering and homogenization	Qualified sequences were clustered into operational taxonomic units (OTUs) at a similarity threshold of 97% use Uparse algorithm of Vsearch (v2.7.1) sofware. To minimize the impact of sequencing depth on inter-sample variations, all sample data were homogenized	
Database comparison	The BLAST tool was used to classify all OUT representative sequences into different taxonomic groups against Silva138 Database, and e-value threshold was set to 1e-5.	The BLAST tool was used to classify all OUT representative sequences into different taxonomic groups against Unite 8.2 Database, and e-value threshold was set to 1e-5.

### 2.5 Data statistics and analysis

The indicators measured in this study and the raw data from microbial high-throughput sequencing were first preliminarily analyzed using Excel 2021, followed by data analysis and graphical visualization using Rstudio software (v4.4.2) and various R packages. Significance tests for differences in various indicators were performed using ANOVA. The R packages used for graphical analysis and visualization in this study were as follows: Venn diagrams using ggVennDiagram 1.5.4, α-diversity analysis using vegan 2.7.1 and ggplot2 3.5.2, PCoA plot creation using vegan 2.7.1, ggrepel 0.9.6 and ggplot2 3.5.2, neutral community model construction and analysis using Hmisc 5.2.3 and minpack.lm 1.2.4, bee colony plots using ggbeeswarm 0.7.2 and ggplot2 3.5.2, trend heatmaps using vegan 2.7.1 and ComplexHeatmap 2.18.0, TOPSIS analysis using plyr 1.8.9 and ggplot2 3.5.2, RDA plots using vegan 2.7.1 and ggplot2 3.5.2; OPLS-DA model and PLS-SEM equation construction, using ropls 1.38.0 and plspm 0.5.1, respectively.

## 3 Results

### 3.1 The effect of different fertilization treatments on tea plant growth

**Figure 1:**
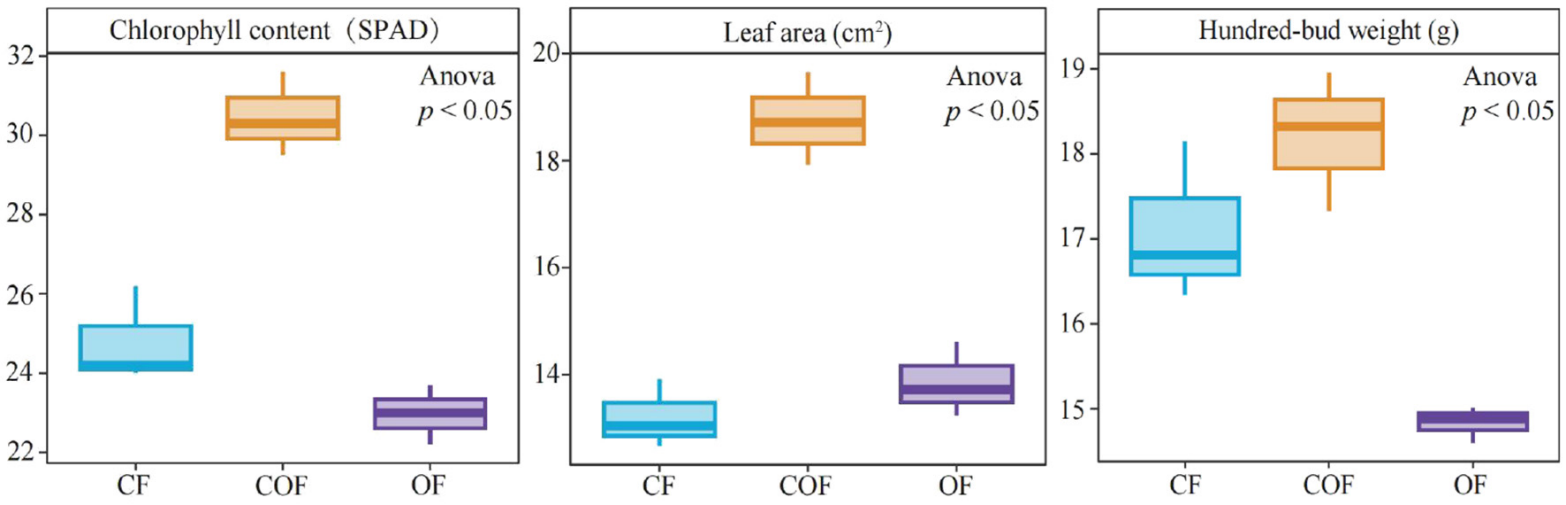
Effects of different fertilization treatments on tea plant growth. CF: 100% chemical fertilizer treatment; COF: 50% chemical fertilizer + 50% organic fertilizer treatment; OF: 100% organic fertilizer treatment. The values in the box plot are expressed as mean ± SD.

This study analyzed the effects of 100% chemical fertilizer (CF), 50% chemical fertilizer + 50% organic fertilizer (COF), and 100% organic fertilizer (OF) treatments on tea plant growth. The results showed (Figure 1) that the chlorophyll content, leaf area, and hundred-bud weight of tea leaves were 24.80 SPAD, 13.21 cm^2^, and 17.10 g under CF treatment; 30.47 SPAD, 18.76 cm^2^, and 18.22 g under COF treatment; and they were 22.97 SPAD, 13.86 cm^2^, and 14.83 g under OF treatment. These findings indicate that compared to the single application of chemical or organic fertilizer, the combined application of chemical and organic fertilizer is more effective in promoting tea plant growth.

### 3.2 Effects of different fertilization treatments on carbon, nitrogen, and phosphorus biomass of soil microorganisms in the rhizosphere of tea plants

**Figure 2:**
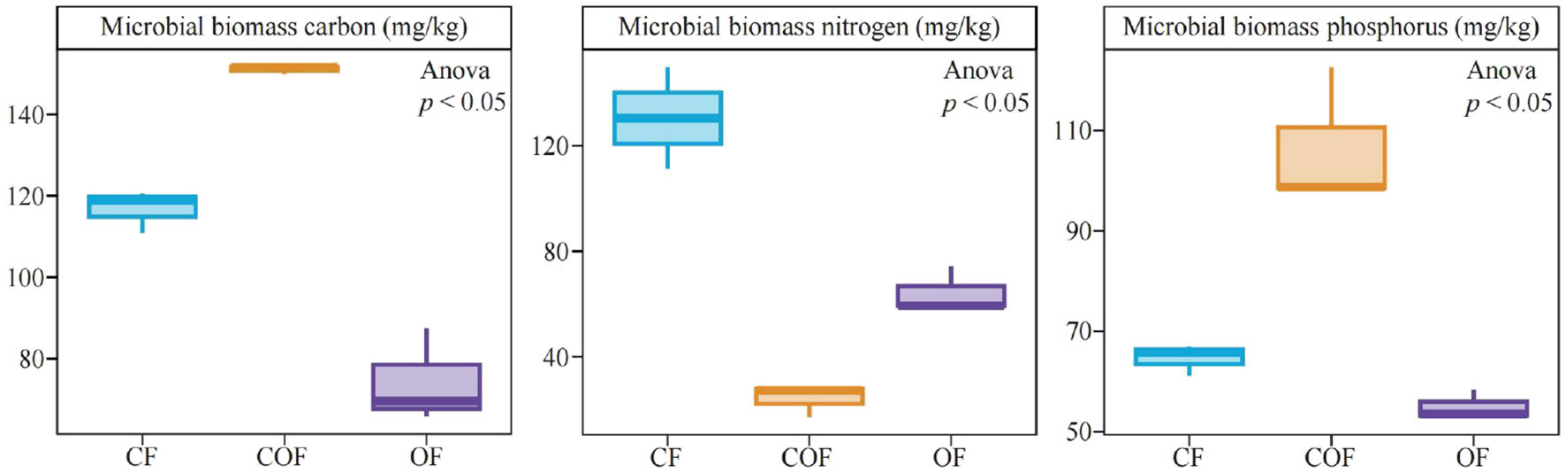
Effects of different fertilization treatments on the biomass carbon, nitrogen, and phosphorus of rhizosphere soil microorganisms of tea plants. CF: 100% chemical fertilizer treatment; COF: 50% chemical fertilizer + 50% organic fertilizer treatment; OF: 100% organic fertilizer treatment. The values in the box plot are expressed as mean ± SD.

Analysis of microbial biomass carbon, nitrogen, and phosphorus in the rhizosphere soil of tea plants after different fertilization treatments (Figure 2) showed that under CF treatment, soil microbial biomass carbon, nitrogen, and phosphorus were 116.76, 130.47, and 64.63 mg/kg, respectively; under COF treatment, these values were 151.05, 24.16, and 105.56 mg/kg, respectively; and under COF treatment, they were 74.30, 64.24, and 54.93 mg/kg, respectively. These results indicate that the single chemical fertilizers application increases soil microbial biomass nitrogen content, while the combined application of chemical and organic fertilizer enhances soil microbial biomass carbon and phosphorus content.

### 3.3 The effect of different fertilization treatments on the diversity of microbial communities in the rhizosphere soil of tea plants

**Figure 3:**
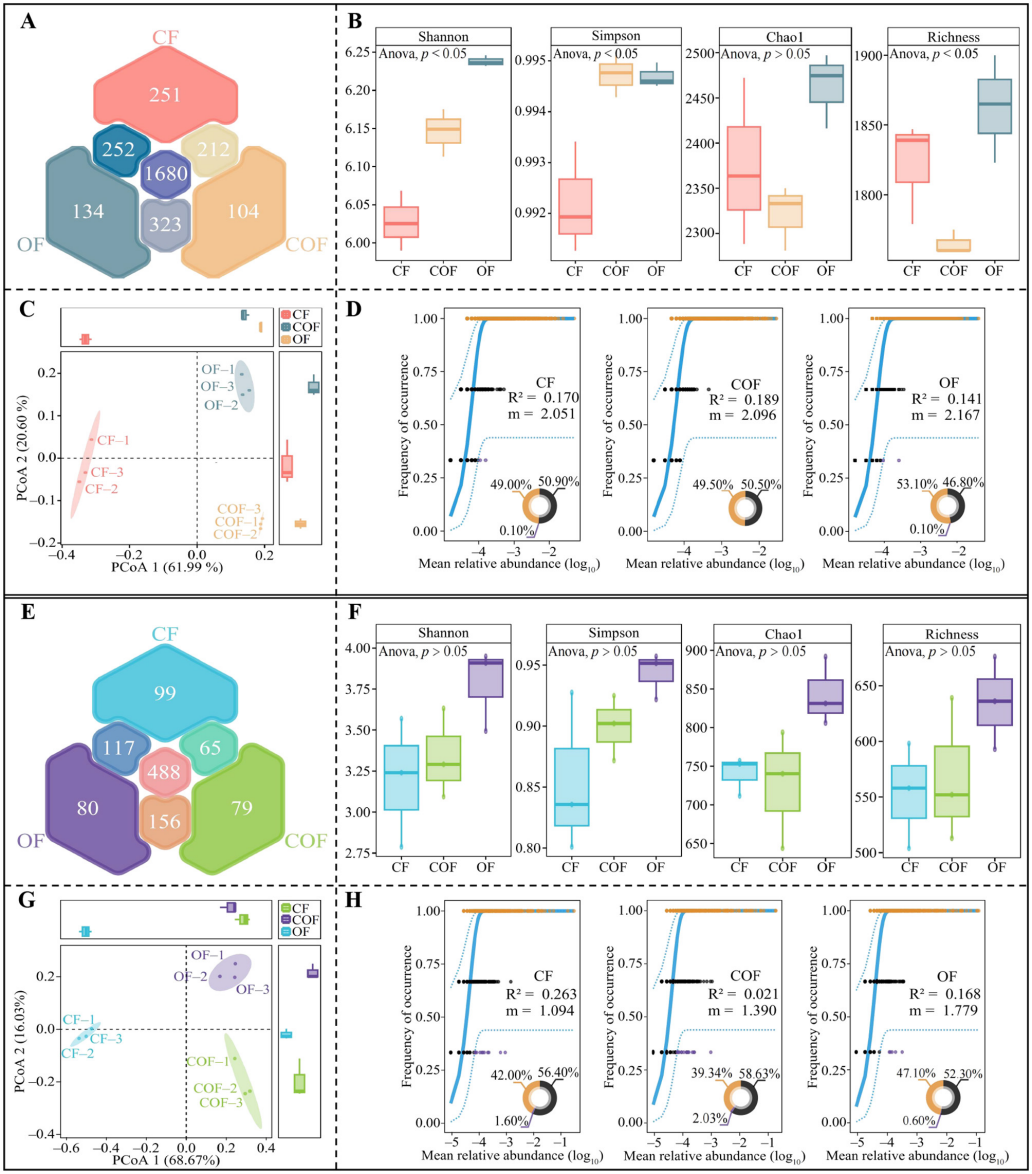
Effects of different fertilization treatments on the structure and diversity of the microbial community in the rhizosphere soil of tea plants. CF: 100% chemical fertilizer treatment; COF: 50% chemical fertilizer + 50% organic fertilizer treatment; OF: 100% organic fertilizer treatment; **(A)** Venn diagram analysis of bacterial community OTUs; **(B)** α-Diversity analysis of bacterial communities; **(C)** PCoA analysis of β-diversity of bacterial communities; **(D)** Neutral community model analysis of bacterial communities; **(E)** Venn diagram analysis of fungal community OTUs; **(F)** α-Diversity analysis of fungal communities; **(G)** PCoA analysis of β-diversity of fungal communities; **(H)** Neutral community model analysis of fungal communities. The values in the box plot are expressed as mean ± SD.

This study employed high-throughput sequencing technology to analyze changes in the microbial community of tea plant rhizosphere soil under different fertilization treatments. Bacterial sequencing results (Figure 3A) revealed a total of 2,956 bacterial OTUs in the rhizosphere soil, with 1,680 OTUs shared among CF, COF, and OF treatments, and 251, 104, and 134 unique OTUs in CF, COF, and OF, respectively. Analysis of bacterial community α-diversity indices (Figure 3B) revealed significant differences among treatments for all indices except Chao1, with the highest α-diversity observed in the OF treatment. PCoA analysis of bacterial community β-diversity (Figure 3C) indicated significant differences across CF, COF, and OF treatments, effectively distinguishing the three treatments into distinct regions. Further analysis using a neutral community model revealed (Figure 3D) that COF treatment exerted the greatest impact on bacterial community structure, as indicated by R^2^ values (COF > CF > OF), while OF treatment had the greatest impact on bacterial community migration rate m values (OF > COF > CF). These results demonstrate that different fertilization treatments significantly influenced the diversity and abundance of bacterial communities in tea plant rhizosphere soil. Compared to the application of a single fertilizer, the combined application of chemical and organic fertilizers had a stronger impact on bacterial community structure, while the sole use of organic fertilizer was more conducive to bacterial community migration and aggregation.

Additionally, fungal sequencing of tea plant rhizosphere soil (Figure 3E) identified a total of 1,084 fungal OTUs, with 488 OTUs shared among CF, COF, and OF treatments, and 99, 79, and 80 unique OTUs in CF, COF, and OF, respectively. Analysis of fungal community α-diversity indices (Figure 3F) showed no significant differences (*p* > 0.05) in the four α-diversity indices (Shannon, Simpson, Chao1, and Richness) across fertilization treatments. β-diversity PCoA analysis (Figure 3G) showed significant differences among CF, COF, and OF, effectively distinguished the three treatments into different regions. Neutral community model analysis revealed (Figure 3H) that CF treatment exerted the greatest impact on fungal community structure, as indicated by R^2^ values (CF > OF > COF), while OF treatment had the greatest impact on fungal community migration rate m values (OF > COF > CF). These results demonstrate that different fertilization treatments significantly influenced the diversity and abundance of fungal communities in the tea plant rhizosphere soil. The single chemical fertilizer application had the greatest impact on fungal community structure, while the single organic fertilizer application was more conducive to fungal community migration and aggregation.

### 3.4 Analysis of microbial screening and quantitative changes in tea plant rhizosphere soil after different fertilization treatments

**Figure 4:**
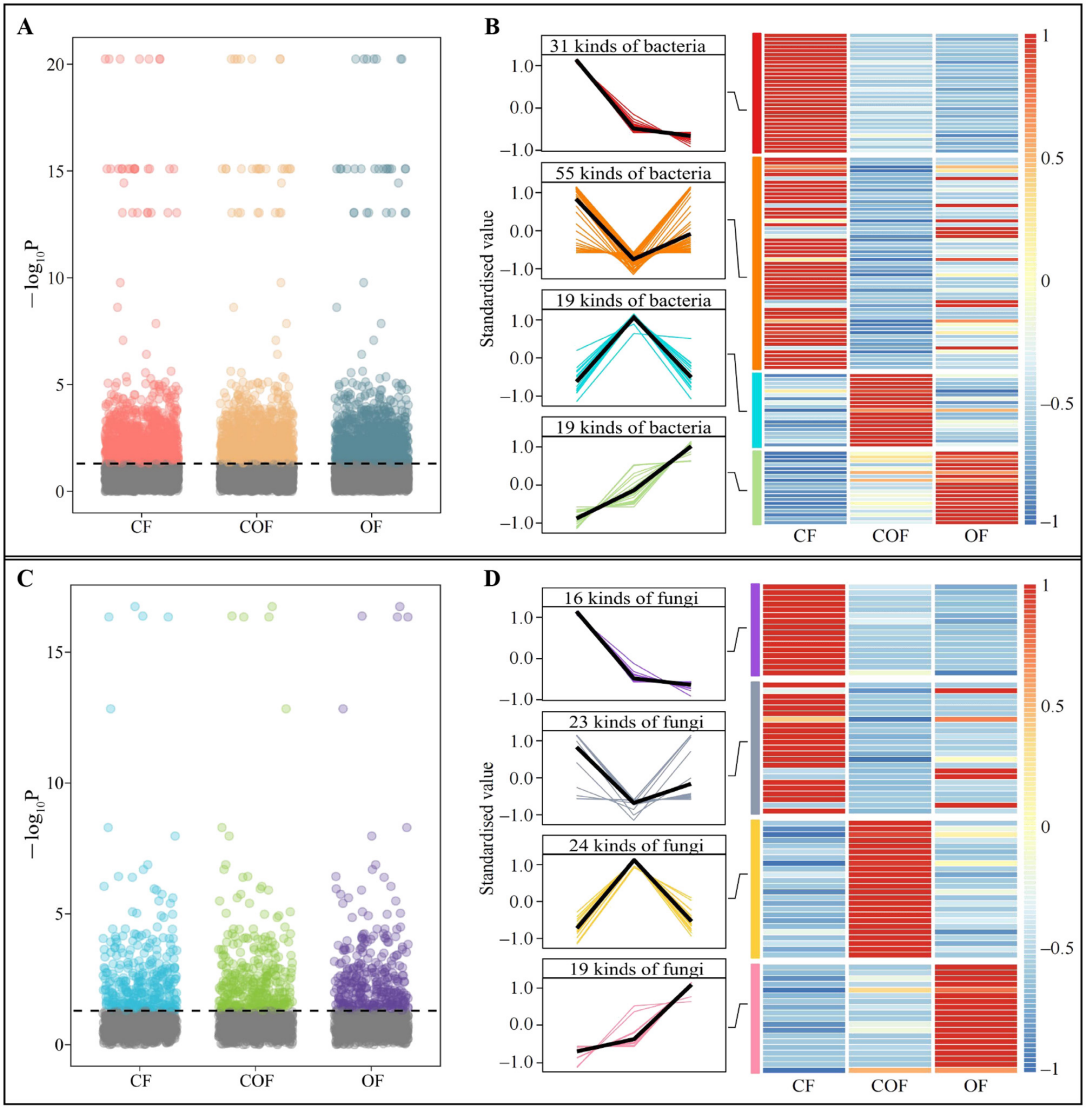
Screening of differential microorganisms and changes in their abundance in tea plant rhizosphere soil after different fertilization treatments. CF: 100% chemical fertilizer treatment; COF: 50% chemical fertilizer + 50% organic fertilizer treatment; OF: 100% organic fertilizer treatment; **(A)** ANOVA test of significant differences in bacterial abundance in tea plant rhizosphere soil after different fertilization treatments; **(B)** Tend analysis of bacterial abundance after different fertilization treatments; **(C)** ANOVA test of significant differences in fungal abundance in tea plant rhizosphere soil after different fertilization treatments; **(D)** Analysis of trends in fungal abundance after different fertilization treatments.

Based on the above analysis, this study further screened for microorganisms with significant differences in the rhizosphere soil of tea plants under different fertilization treatments. ANOVA analysis of bacterial communities (Figure 4A) identified 992 bacterial OTUs with significant differences among CF, COF, and OF. Based on OTU annotation results, the trends in bacterial abundance (Figure 4B), revealed that the abundance trends of 124 bacterial genera could be categorized into four main patterns: CF > COF > OF (31 genera), CF > OF > COF (55 genera), COF > OF > CF (19 genera), and OF > COF > CF (19 genera). Furthermore, ANOVA test of fungal community OTUs (Figure 4C) identified 316 OTUs with significant differences among the three treatments, which were annotated to 82 fungal genera. Among 82 fungal genera, 16 genera showed an abundance trend of CF > COF > OF, 23 genera CF > OF > COF, 24 genera COF > OF > CF, and 19 genera OF > COF > CF (Figure 4D). These findings demonstrate that different fertilization treatments induced significant changes in the structure and abundance of microbial communities in the tea plant rhizosphere, with distinct effects on soil microorganisms across treatments.

### 3.5 Analysis of key microbial screening and quantitative changes in tea plant rhizosphere soil after different fertilization treatments

**Figure 5:**
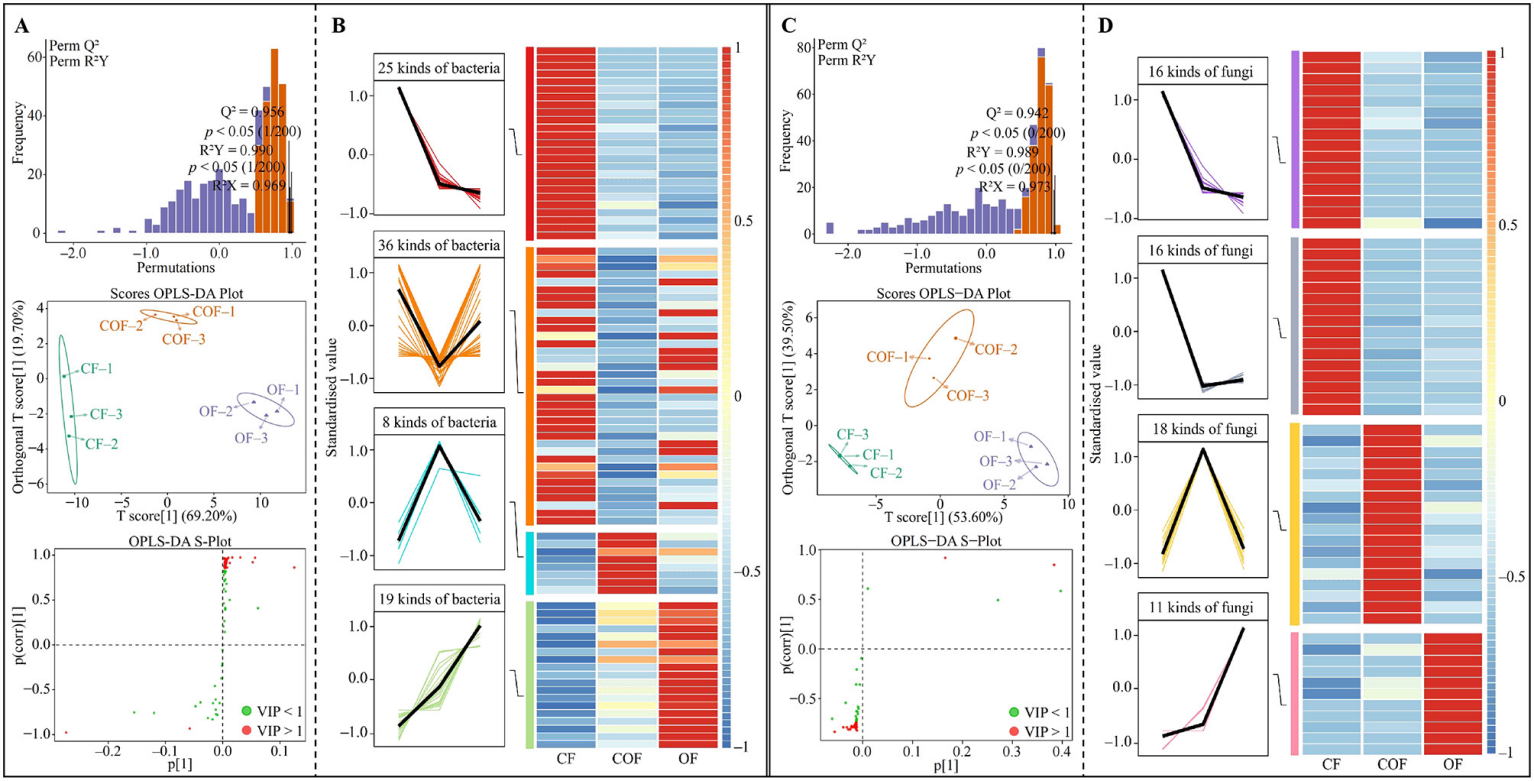
Screening and analysis of quantity changes in key differential microorganisms within tea rhizosphere soil following various fertilization treatments. CF: 100% chemical fertilizer treatment; COF: 50% chemical fertilizer + 50% organic fertilizer treatment; OF: 100% organic fertilizer treatment; **(A)** Construction of OPLS-DA models for CF, COF, and OF based on differential bacteria to identify key differential bacteria; **(B)** Trend analysis of variations in the abundance of key differential bacteria following different fertilization treatments; **(C)** Construction of OPLS-DA models for CF, COF, and OF based on differential fungi to identify key differential fungi; **(D)** Trend analysis of variations in the abundance of key differential fungi following different fertilization treatments.

The above analysis has uncovered significant alterations in the structure and abundance of microbial communities within the rhizosphere soil of tea plants following various fertilization treatments. Building upon this, this study further developed OPLS-DA models for CF, COF, and OF, utilizing microorganisms exhibiting notable differences to identify key differential microorganisms. The outcomes of the OPLS-DA model construction for bacterial communities in CF, COF, and OF (Figure 5A) demonstrated that both the model's goodness of fit (R^2^Y) and predictive power (Q^2^) achieved statistically significant levels (*p* < 0.05). The key bacteria (VIP > 1) that distinguished different fertilization treatments belonged to 88 genera. After an in-depth analysis of the trends in the number of key bacteria (see Figure 5B), it was observed that the numbers of the 25 bacterial genera exhibited a trend of CF > COF > OF; for the 36 genera, the trend was CF > OF > COF; 8 genera demonstrated a trend of COF > OF > CF; and 19 genera showed OF > COF > CF. Furthermore, when constructing the OPLS-DA model for fungal communities based on CF, COF, and OF (Figure 5C), the results indicated that both R^2^Y and Q^2^ of the model reached significant levels, effectively differentiating between CF, COF, and OF. Notably, the key fungi from 61 genera played a pivotal role in distinguishing these three treatments. Upon further analysis of the trends in the number of key fungi (Figure 5D), it was revealed that among the 16 fungal genera, the numbers exhibited a trend of CF > COF > OF; 16 fungal genera showed CF > OF > COF; 18 fungal genera demonstrated a trend of COF > OF > CF; and 11 fungal genera showed OF > COF > CF. Evidently, different fertilization treatments exert a significant influence on the microbial community structure and abundance in tea plant rhizosphere soil, particularly among key microorganisms, which may further impact the functions of these microorganisms.

### 3.6 Functional analysis of key microbial differences in the rhizosphere soil of tea plants following various fertilization treatments

**Figure 6:**
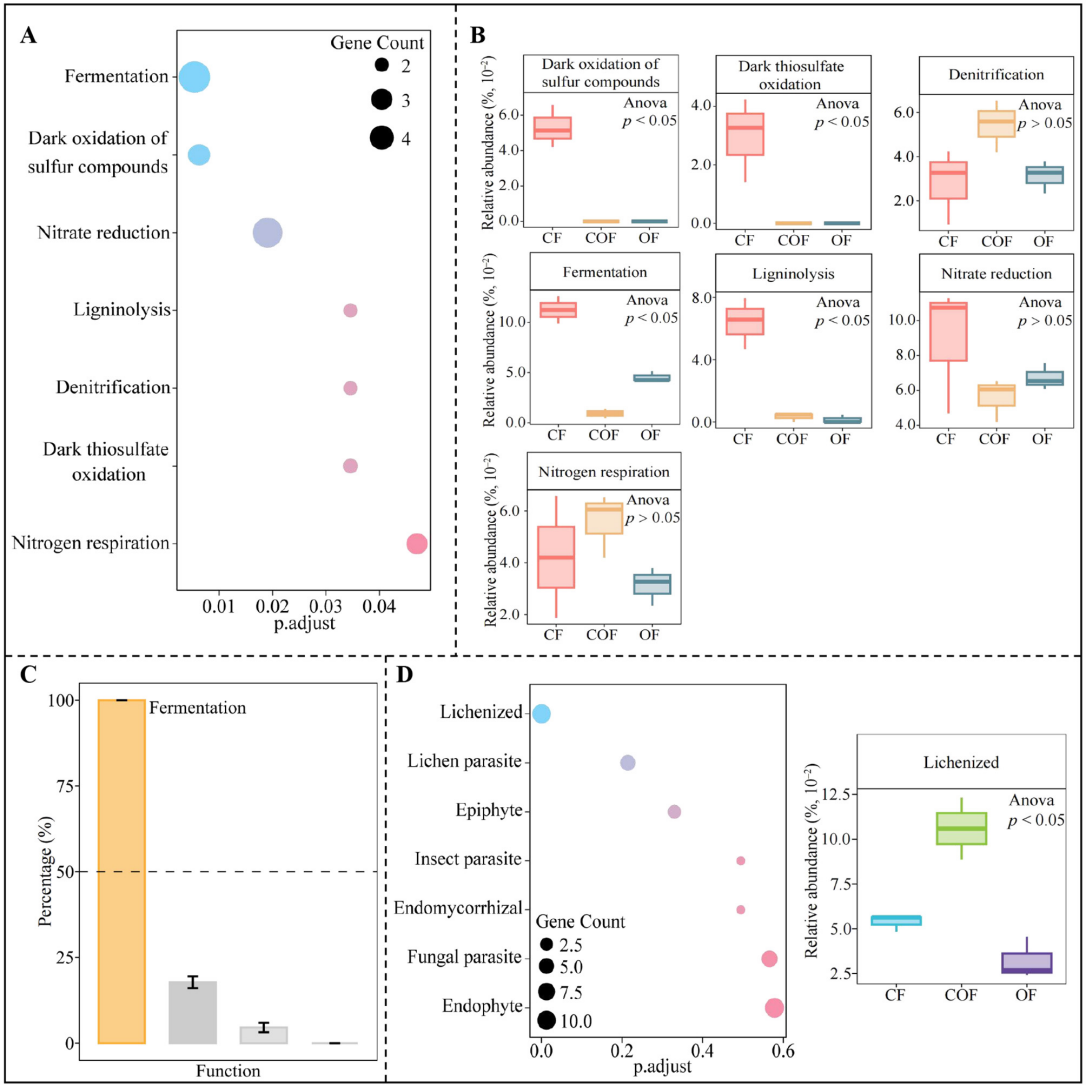
Functional and intensity analysis of key differential microorganisms in the rhizosphere soil of tea plants under different fertilization treatments. CF: 100% chemical fertilizer treatment; COF: 50% chemical fertilizer + 50% organic fertilizer treatment; OF: 100% organic fertilizer treatment; **(A)** Functional enrichment of key bacteria; **(B)** The intensity analysis of functions significantly enriched by key bacteria; **(C)** TOPSIS analysis of the contribution rate of significantly enriched bacterial functions in distinguishing different fertilization treatments to screen for key functions; **(D)** Functional enrichment of key fungi and the intensity analysis of significantly enriched functions. The values in the box plot are expressed as mean ± SD.

Based on the above analysis, 88 genera of key bacteria and 61 genera of key fungi were selected for functional prediction and enrichment to investigate the impact of various fertilization treatments on the functions of key microorganisms in tea rhizosphere soil. Notably, the key bacteria were significantly enriched in 7 functional categories (Figure 6A). Analysis of functional intensity (Figure 6B) revealed significant differences in the intensity of 4 functions predominantly enriched by the key bacteria under different fertilization treatments, with the highest intensity observed under CF treatment for dark oxidation of sulfur compounds, dark thiosulfate oxidation, fermentation, and ligninolysis. Further analysis using TOPSIS to assess the contribution of these 4 significantly different functions in distinguishing between CF, COF, and OF (Figure 6C) showed that only fermentation had a contribution rate exceeding 50%. Additionally, this study further predicted and enriched the functions of key fungi (Figure 6D), resulting in only enriching one function, i.e., lichenized. The functional intensity analysis indicated that the function intensity of lichenized was highest under COF treatment. It is evident that different fertilization treatments altered the abundance of key microorganisms in the rhizosphere soil of tea plants, which subsequently influenced the functions of soil microorganisms, particularly fermentation and lichenized.

### 3.7 Analysis of the sources and quantities of key functional microorganisms under different fertilization treatments, and screening of characteristic microorganisms

**Figure 7:**
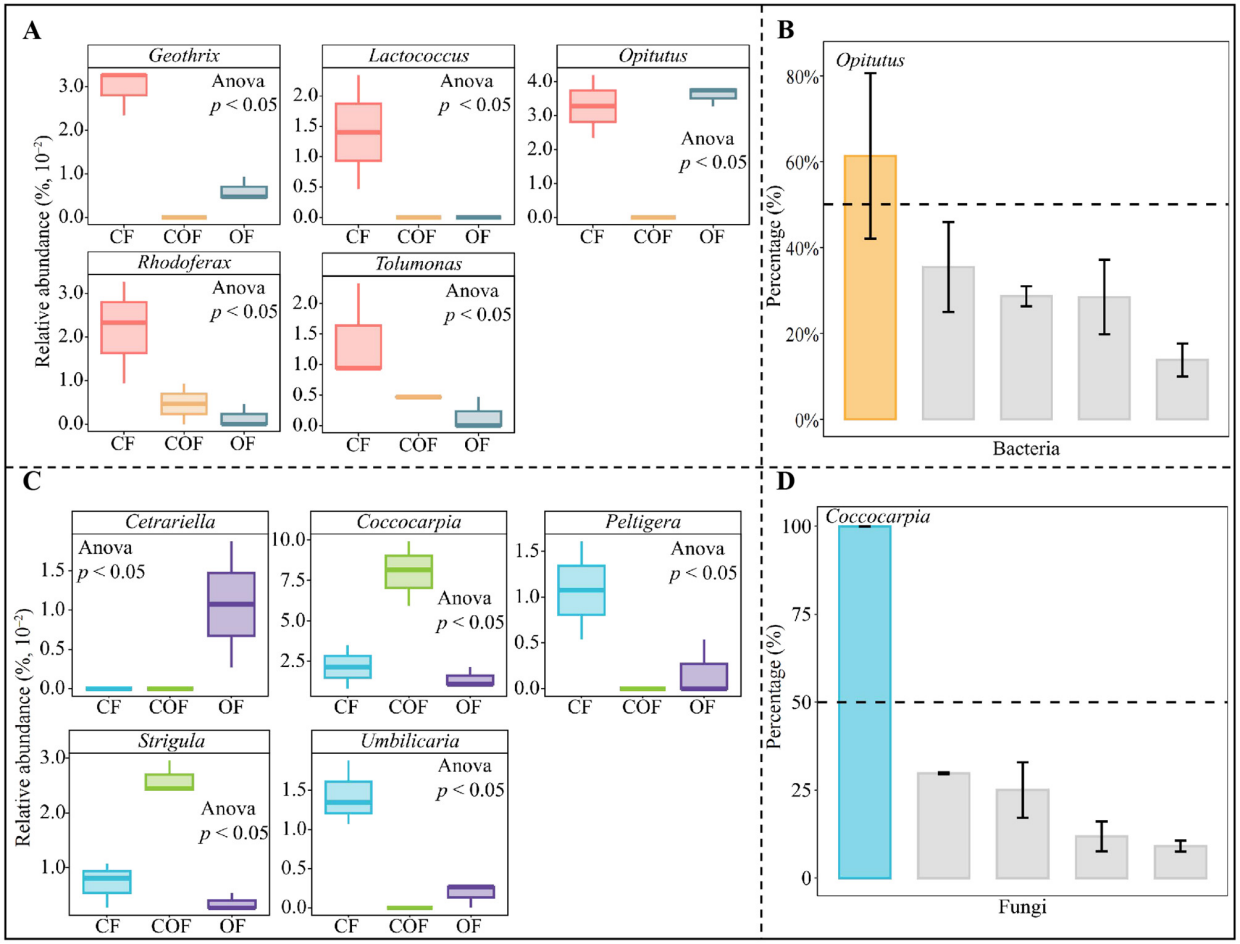
Analysis of the sources and quantities of key functional microorganisms under different fertilization treatments, and screening of characteristic microorganisms. CF: 100% chemical fertilizer treatment; COF: 50% chemical fertilizer + 50% organic fertilizer treatment; OF: 100% organic fertilizer treatment; **(A)** Analysis of the microbial sources and quantities of key bacterial functions; **(B)** TOPSIS analysis of the contribution rates of functional bacteria in distinguishing different fertilization treatments to obtain characteristic bacteria; **(C)** Analysis of the microbial sources and quantities of key fungal functions; **(D)** TOPSIS analysis of the contribution rates of functional fungi in distinguishing different fertilization treatments to obtain characteristic fungi. The values in the box plot are expressed as mean ± SD.

Building on the key function of tea plant rhizosphere soil microorganisms identified under different fertilization treatments (described above), this study further traced the microbial sources underlying these critical functions. As shown in Figure 7A, the fermentation function primarily originated from 5 bacterial genera, i.e., *Geothrix, Lactococcus, Opitutus, Rhodoferax* and *Tolumonas*. Notably, the abundance of these five genera varied significantly across fertilization treatments. TOPSIS analysis was used to determine the contribution rate of the 5 bacterial genera to fermentation function (Figure 7B), and found that among the 5 key bacterial genera, only *Opitutus* contributed more than 50% to fermentation function. Additionally, the lichenized function was found to derive mainly from five fungal genera, including *Cetrariella, Coccocarpia, Peltigera, Strigula*, and *Umbilicaria* (Figure 7C). Similar to the bacterial genera, the abundance of these fungal genera differed significantly among fertilization treatments. TOPSIS-based assessment of the contribution of the 5 fungal genera to lichenized function (Figure 7D) indicated that only *Coccocarpia* exceeded a 50% contribution rate. These results collectively suggest that different fertilization treatments primarily influenced the abundance of *Opitutus* (bacteria) and *Coccocarpia* (fungi)—key microorganisms in the tea plant rhizosphere—thereby altering microbial fermentation and lichenized functions, which in turn may impact tea plant growth.

### 3.8 Interactions between characteristic microorganisms and their functions and various indicators

**Figure 8:**
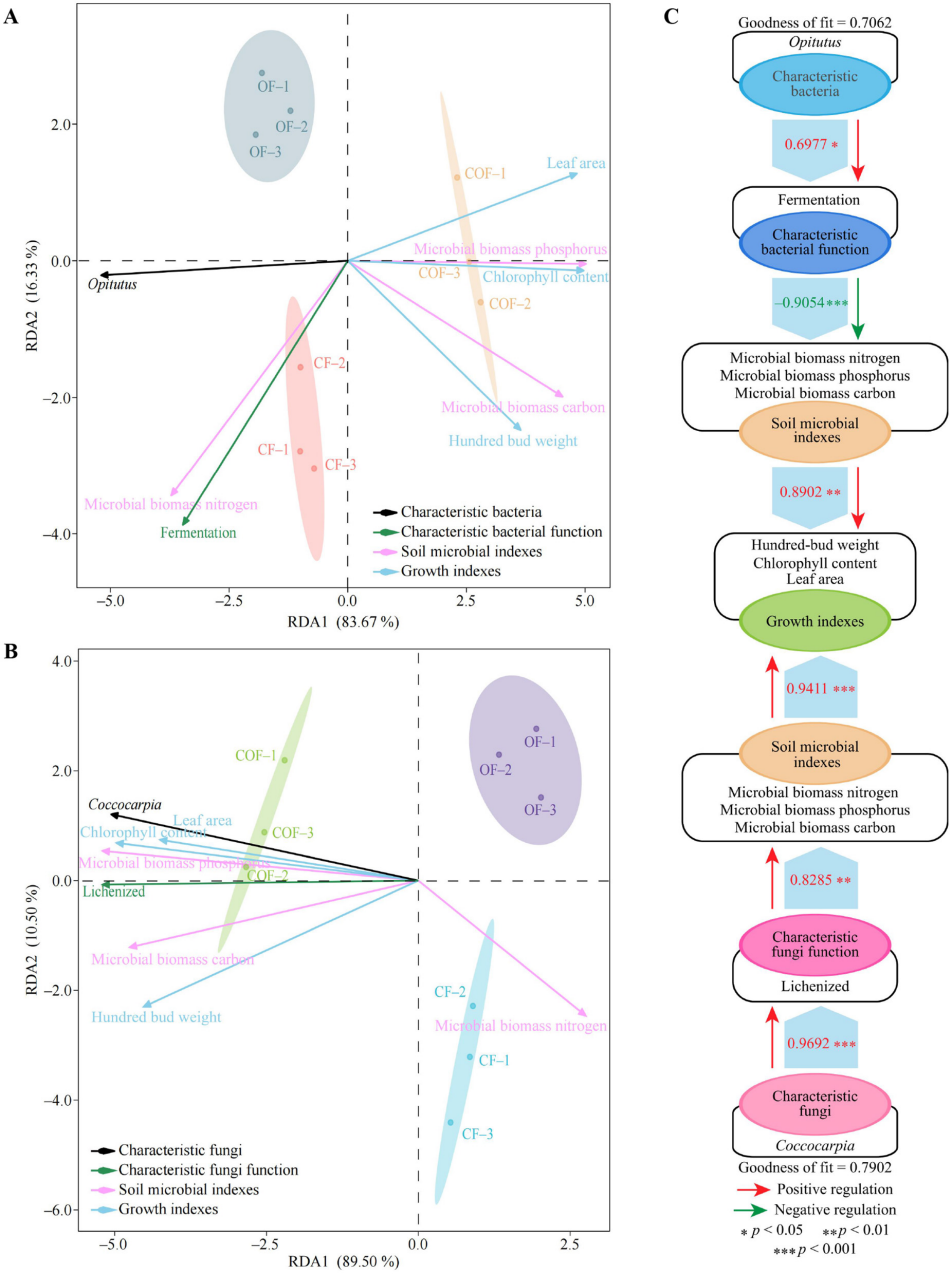
Interactions between characteristic microorganisms and their functions and different indicators. CF: 100% chemical fertilizer treatment; COF: 50% chemical fertilizer + 50% organic fertilizer treatment; OF: 100% organic fertilizer treatment; **(A)** RDA analysis of characteristic bacteria and their functions with different indicators; **(B)** RDA analysis of characteristic fungi and their functions with different indicators; **(C)** PLS-SEM equation construction of characteristic microorganisms and their functions with different indicators.

This study further explored the interactive relationships between characteristic microorganisms and their functions and various indicators. The RDA analysis results of characteristic bacteria and their functions with different indicators (Figure 8A) showed that the indicators mainly related to CF and OF were the characteristic bacteria *Opitutus*, fermentation function, and microbial biomass nitrogen, while other indicators were mainly related to COF. The RDA analysis results of characteristic fungi and their functions with different indicators (Figure 8B) indicated that the indicators primarily associated with CF and OF were microbial biomass nitrogen, while other indicators were primarily associated with COF, including the characteristic fungus *Coccocarpia* and lichenized function. To further analyze these relationships, this study constructed PLS-SEM equations for different indicators (Figure 8C). The results showed that the characteristic bacterium *Opitutus* positively regulated fermentation function intensity (0.70^*^), fermentation function negatively regulated soil microbial biomass carbon, nitrogen, and phosphorus (−0.91^***^), and soil microbial biomass carbon, nitrogen, and phosphorus positively regulated tea plant growth indicators (0.89^**^). Additionally, the characteristic fungus *Coccocarpia* positively regulated the intensity of lichenized function (0.97^***^), positively regulated soil microbial biomass carbon, nitrogen, and phosphorus (0.83^**^), and positively regulated tea plant growth indicators (0.94^***^). It is evident that increasing the abundance of the characteristic fungus *Coccocarpia* in the rhizosphere soil of tea plants and reducing the abundance of the characteristic bacterium *Opitutus* can enhance soil lichenized function, reduce fermentation function, and promote tea plant growth.

## 4 Discussion

Fertilization is a critical agronomic practice in tea cultivation, with the choice of fertilization patterns directly influencing tea plant growth and development (Xie et al., 2022). Against the backdrop of the current strong advocacy for ecological agriculture, exploring fertilization patterns that substitute organic fertilizers for chemical fertilizers in tea plantations has emerged as a research hotspot and important direction in tea cultivation (Que and Zhao, 2024). This study compared the effects of different fertilization treatments on tea plant growth and found that under COF treatment, chlorophyll content, leaf area, and hundred-bud weight were significantly higher than those under CF and OF treatments. In general, organic fertilizers improve soil structure and enhance nutrient utilization efficiency, while chemical fertilizers can rapidly supply plants with key elements such as nitrogen, phosphorus, and potassium, and their combination can synergistically promote crop growth (Li et al., 2021; Imran, 2024). Additionally, the humic acid and various microbial metabolites abundant in organic fertilizers may stimulate the development of tea plant root systems, thereby further enhancing their nutrient absorption efficiency (Chen et al., 2021). However, in this study, tea plants receiving sole organic fertilizer exhibited poorer growth indicators, which may be attributed to the slow nutrient release rate of organic fertilizer. Since the mineralization rate of organic fertilizer is significantly influenced by environmental factors such as temperature and humidity, it may fail to meet the short-term nutrient demands of rapidly growing tea plants (Shaji et al., 2021). Conversely, while single chemical fertilizers can temporarily increase soil available nutrients, prolonged use may lead to soil acidification and microbial community imbalance, thereby affecting tea plant growth (Manzoor et al., 2024). It is evident that the combined application of chemical fertilizers and organic fertilizers can leverage their respective advantages while mitigating their drawbacks, thereby more effectively promoting tea plant growth.

The growth of tea plants is closely tied to the supply of soil nutrients, and soil microbial biomass carbon, nitrogen, and phosphorus are key indicators for evaluating soil fertility. This study found that under COF treatment, the microbial biomass carbon and phosphorus in the rhizosphere soil of tea plants were significantly higher than those under CF and OF treatments, while the microbial biomass nitrogen was highest under CF treatment. This result may be related to the impact of different fertilization patterns on microbial nutrient utilization strategies. Chemical fertilizers can directly increase soil inorganic nitrogen content, promoting the growth of microorganisms primarily involved in nitrogen metabolism (Sindhu et al., 2024), while organic fertilizers can stimulate the proliferation of microorganisms involved in carbon fixation and phosphorus activation by providing carbon and phosphorus sources (Wu et al., 2024). It is evident that differences in fertilization patterns may directly influence the community structure of soil microorganisms.

Therefore, this study further investigated the effects of different fertilization patterns on the structure and function of the microbial community in the rhizosphere soil of tea plants. The results showed that after OF treatment, the bacterial and fungal migration rates and α-diversity in the rhizosphere soil of tea plants were the highest. COF had the greatest impact on bacterial community structure, while CF had the greatest impact on fungal communities. It has been reported that organic fertilizers have a rich composition, which helps enhance microbial dispersal capacity, promote community dynamic balance, and thereby improve soil texture (Xing et al., 2025). The use of chemical fertilizers significantly reduces bacterial community diversity in soil but promotes fungal reproduction, increasing fungal abundance (Yang et al., 2024). The combined application of organic and chemical fertilizers helps balance the structure and function of bacterial and fungal communities in soil, thereby more effectively promoting plant growth (Xiao et al., 2025). It is evident that fertilization patterns alter the microbial community structure in tea plant rhizosphere soil, which may in turn affect the function of certain key microorganisms and influence tea plant growth.

Therefore, this study further screened the characteristic microorganisms in the rhizosphere soil of tea plants under different fertilization patterns and analyzed their functions. The results indicated that different fertilization treatments primarily affected the abundance of *Opitutus* and *Coccocarpia* of the key microorganisms in the rhizosphere soil of tea plants, thereby influencing their fermentation and lichenized functions, which in turn affected tea plant growth. Secondly, COF treatment had the lowest in the *Opitutus* population in the tea plant rhizosphere soil, with the weakest fermentation function, while the *Coccocarpia* population was the highest, with the strongest lichenized function. It has been reported that *Opitutus* can degrade lignocellulose, has weak tolerance to oxygen, and is better adapted to anaerobic and microaerophilic conditions (Muszynski et al., 2021). Fermentation metabolic pathways are typically associated with anaerobic decomposition of organic matter but contribute little to soil nutrient cycling (Möller, 2015; Zhou et al., 2018). *Coccocarpia* plays an important role in ecosystems, and the enhancement of its lichenized function can increase photosynthesis and nitrogen fixation capacity, providing carbon and nitrogen sources for plant growth (Cameron et al., 2024). Additionally, the secondary metabolites it secretes have antioxidant and pathogen-resistant properties, promoting plant growth (Charria-Girón et al., 2023). This study found that *Opitutus* positively regulated fermentation function intensity but was detrimental to the accumulation of carbon, nitrogen, and phosphorus in soil microbial biomass, thereby hindering tea plant growth. In contrast, *Coccocarpia* positively regulated lichenized function intensity, which enhanced the content of carbon, nitrogen, and phosphorus in soil microbial biomass and promoted tea plant growth. It is evident that increasing the abundance of *Coccocarpia* of the characteristic fungus in tea plant rhizosphere soil and reducing the abundance of *Opitutus* of the characteristic bacterium can enhance soil lichenized function, reduce fermentation function, and promote tea plant growth.

## 5 Conclusion

**Figure 9:**
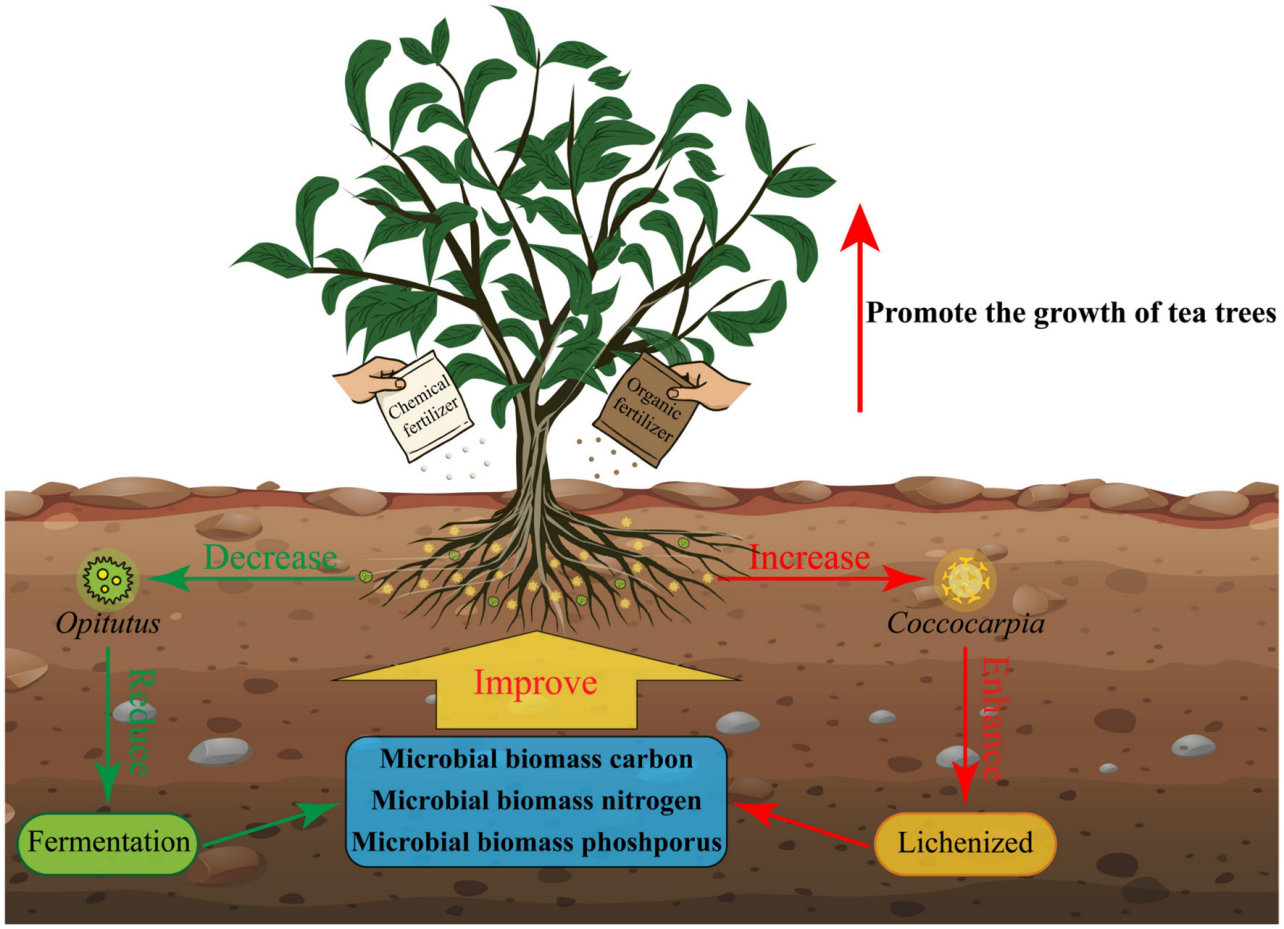
The application of chemical fertilizers and organic fertilizers regulates the number and function of characteristic microorganisms in the rhizosphere soil of tea plants, thus affects the growth of tea plants.

This study analyzed the effects of different fertilization treatments on tea plant growth, rhizosphere soil microbial biomass, community diversity, and function. The results showed (Figure 9) that compared with the single fertilizer application, the combined application of chemical fertilizer and organic fertilizer effectively increased the relative abundance of the characteristic fungus *Coccocarpia*, while reducing that of the characteristic bacterium *Opitutus* in the rhizosphere soil. These microbial abundance shifts enhanced the soil's lichenized function, reduced fermentation function, and significantly increased the microbial biomass carbon, nitrogen, and phosphorus contents, ultimately promoting tea plant growth. This study preliminarily explored the effects of fertilizer ratios on the rhizosphere soil microorganisms and tea tree growth. In the future, further exploration is needed to determine the optimal fertilizer ratio in tea plantation fertilization to achieve the best fertilization effect. The results of this study provide a theoretical basis for regulating soil microbial community structure and function through scientific fertilization in tea plantations, thereby optimizing tea plant growth.

## Data Availability

The data presented in this study are publicly available. This data can be found here: https://www.ncbi.nlm.nih.gov/sra, accession PRJNA1300590.
